# Use of cross-reactivity immunoassay to orient insulin replacement in diabetic patients with high levels of insulin antibodies

**DOI:** 10.1016/j.mex.2016.08.003

**Published:** 2016-08-05

**Authors:** Alejandro Cardoso Landaburu, María Pomares, Alfredo Avalos, Silvia Lapertosa, Gustavo Frechtel, Edgardo Poskus

**Affiliations:** a Universidad de Buenos Aires, Facultad de Farmacia y Bioquímica, Cátedra de Inmunología-IDEHU (UBA-CONICET), Buenos Aires, Junín 956, CABA, CP C1113AAD, Argentina; b Servicio de Endocrinología y Diabetes del Hospital Juan Pablo II, San Martin 569 Corrientes Capital CP 3400, Argentina; c Servicio de Endocrinología, Nutrición y Diabetes, Hospital J. R. Vidal, Necochea 1050, Corrientes Capital CP 3400, Argentina; d Servicio de Nutrición, Departamento de Medicina, Facultad de Medicina, Universidad de Buenos Aires (UBA), Paraguay 2155, CABA C1121ABG, Argentina

**Keywords:** Radio binding assay (RBA), Radio immuno assay (RIA), Insulins analogues, Diabetes type 1, Insulin antibodies, Brittle diabetes

## Abstract

The prevalence and high levels of anti-insulin antibodies (IA) have frequently been associated with brittle diabetes, lipodystrophy in the areas where the insulin is injected and/or poor metabolic control. When this happens the usual criterion adopted is the empirical change of insulin type and/or formulation intending to diminish the IA level and then to decrease the undesirable side-effects. Here, we present a rational two step radiometric method consisting in: A) a first-line radioligand binding assay (RBA) to assess IA in sera of these patients and detecting those with high levels. B) applying a displacement assay (RIA) to determine the in vitro cross-reactivity parameters (affinity constants and selectivity ratios) that quantify the relative degree of interaction between antibodies and alternative insulin analogs. From these results we conclude that conventional criteria for selection of insulin analogs, in terms of pharmacokinetic and pharmacodinamic parameters, should be complemented with an appropriate test to assess affinity parameters when high IA title is demonstrated.

• This manuscript introduces a rational method to determine the appropriated insulin replacement when high insulin antibodies levels are present.
• This protocol provides instructions and details in mathematical tools and laboratory processes for the analysis of serum samples.
• This method proved to be successful in a single case and requires confirmation using a large group of patients.

## Graphical abstract

Buffer P/G/BSA: 0,1 M Phosphate, 0,25% of non specific gamma globulin and 0,5% of bovin serum albumin, pH 7,4. Veronal Buffer: 0,05 M sodium barbital and 0,01% tween 20, pH 8,6. PEG: Polyethylene glycol 6000. A) RBA: IA binding rate measured as tracer binding percent (B%) over a cutoff of nonspecific binding. B) RIA: B and F results are transformed in plots of B/F = f (ligand dose, M) to calculate the respective K_0_ values. The molar concentration of the tracer in the test must be lower than the inverse of K_0_ value. This condition precludes the preparation of the respective labeled competitors to perform specific single RIAs for each homologous ligand (Berzofsky-Schechter [1]).

**Figure:**
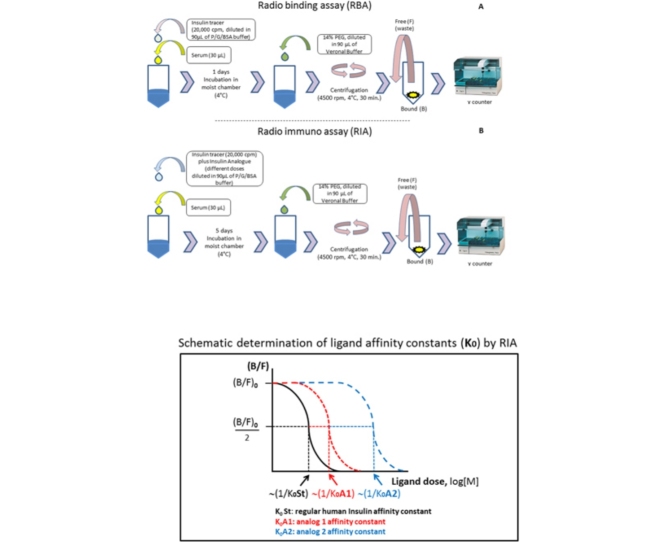

## Introduction

The prevalence and levels of anti-insulin antibodies (IA) elicited nowadays during insulin therapy have decreased remarkably in comparison to the original treatments as a consequence of the high purity of recombinant human insulin preparations and hypoimmunogenicity of insulin analogs. However, some patients still present relatively high IA titer levels, frequently associated with brittle diabetes, lipodystrophy in injection sites and/or poor metabolic control despite the high insulin doses administered. When this happens the usual criterion adopted is the empirical change of insulin type and/or formulation intending to diminish the IA level and then to decrease the undesirable side-effects. However, the consequent problem is that such an empirical procedure requires relatively long periods of time and clinical surveillance during each interval of change, whilst immediate and long-term complications may persist or aggravate.

Here, we present a rational method supporting the best choice of alternative insulin variants according to the immune cross-reactivity parameters exhibited by a panel of candidate formulations assayed in vitro with serum from patients who present with a high positive IA titer. We report the preliminary results obtained after applying the approach mentioned above to a unique model sample consisting of a high level IA positive serum from a diabetic patient exhibiting persistent poor metabolic control despite the high dose of regular and NPH insulin administered.

## Methods details

### Patient

The patient selected for this study was a 69 year old woman who presented type 2 diabetes, diagnosed 10 years ago and treated with insulin only for the last two years. The study followed the recommendations of WMA Declaration of Helsinki [2]. She was admitted to hospital (Hospital J. R. Vidal, Corrientes City, R. Argentina) presenting with hyperglycaemia [U1] and inadequate metabolic control despite treatment with more than 3 IU/kg/day of NPH human insulin (Humulin – Eli Lilly) complemented with crystalline human insulin during the last two years. Moreover, frequently the glycaemia reached values as high as 500 mg/dL (27.7 mmol/L) and HbA_1C_ values of 11% (IFCC 97 mmol/mol). During such episodes circulating insulin antibodies (IA) were assessed by using conventional radioligand binding assay (RBA) and then displacement radioimmunoassay (RIA) for cross-reactivity tests applied to insulin and insulin analogs, both based on slight modifications of previously described methods as follows.

#### Production of the homogeneous tracer mono [^125^I A14] insulin

The homogeneous mono [^125^I A14] Insulin used as a tracer for both radiometric methods, RBA and RIA, was produced using recombinant human insulin (HumulinC from Eli Lilly, Indiana, USA) devoid of preservative substances by chromatography on a Sephadex G25 column (Amersham Biosciences, New Jersey, USA). The labeling and HPLC purification steps were carried out following as indicated by Linde et al. [3] and Jørgensen et al. [4], respectively. Specific radioactivity of roughly 300–380 μCi/μg was routinely achieved.

The concentration of tracer in both, RBA and RIA, was 5.8 × 10^−11^, calculated from an activity of 20,000 cpm; Relative mass of insulin, 5808 Da; Final incubation volume, 0,12 mL; Gamma counter efficiency, 70%; Specific activity of fresh insulin tracer, 380 μCi/μg.

#### IA assessment by RBA

Radioligand binding assay for IA assessment, performed routinely as official analysis service at IDEHU-CONICET laboratory, was based on the original procedure indicated by Kurtz et al. [5] and carried out essentially as previously described by Stumpo et al. [6]. Briefly, duplicate serum aliquots of 30 μL were incubated for 1 days at 4 °C with 90 μL of 0,1 M Phosphate, 0,25% of non specific gamma globulin and 0,5% of bovin serum albumin, pH 7,4 containing per tube 20,000 cpm mono ^125^I[A14] Insulin obtained as indicated above. For each serum sample assayed additional duplicate tubes with an excess of unlabeled human insulin (7.5 × 10^−6^ mol/l) were used to evaluate non-specific binding. The bound insulin fraction was then precipitated and separated using 1 mL of 14% polyethylene glycol 6000 (PEG) in veronal Buffer: 0,05 M sodium barbital and 0,01% tween 20, pH 8,6 (Graphical abstract, panel A). Results were expressed as specific insulin binding percentage B% = Bwi% (preincubated without cold insulin) – Bie% (preincubated with excess cold insulin). Reproducibility was assessed at different binding levels. In a typical routine control test, intra-assay CVs were 5 and 6% for binding values of 15 and 45% (n = 10), respectively, while the corresponding values for inter-assay CVs were 14 and 8% (n = 10), respectively.

To obtain the RBA cutoff value 30 normal human sera were used. Bound, B%, signals were normally distributed and an assay was considered positive if such signal was over the mean plus 3 SD (habitually ∼4%).

The RBA procedure for insulin antibodies (mainly autoantibodies to insulin, IAA) was externally controlled by international proficiency tests (Immunology Diabetes Workshop, University of Florida, USA) since 1996, in which the laboratory typically achieved the following scores: 82% sensitivity, 82% validity, and 100% specificity and consistency for IAA. After this, the laboratory participated in other quality international programs (DASP, IASP) including IAA and other type 1 diabetes marker assessment.

#### RIA and data processing for affinity and cross-reactivity determinations

For in vitro cross-reactivity assessment a displacement RIA was applied by using the tracer described above. Briefly, duplicate 30 μL serum samples from the patient were incubated during 5 days at 4 °C (near equilibrium) with a constant amount of tracer (∼20,000 cpm). Parallel displacement experiments were carried out by using increasing amounts, in duplicates, of the following antigens: unlabeled regular crystalline human insulin (Humulin^®^, Eli Lilly), and the insulin analogs Detemir (Levemir^®^, Novo Nordisk), Glargine (Lantus^®^, Sanofi Aventis), Glulisine (Apidra^®^, Sanofi Aventis) and Lispro (Humalog^®^, Eli Lilly), in doses ranging from 1 × 10^−9^ to 10^−6^ M (Graphical abstract, panel B). Insulin analogs were engineered to enhance desired molecular properties without altering immunogenicity, at least in theory. However, since the amino acid sequence of these insulin variants were changed to alter its absorption, distribution, metabolism and excretion characteristics, some residual capacity to elicit specific antibodies must be expected. This is the case if high sensitivity radioimmunoassays as those described here are used to detect circulating IA. Also the grade of alteration of the natural insulin molecule is of importance.

Detemir is an insulin analog in which a fatty acid (myristic acid) is bound to the lysine amino acid at position B29. This molecule has a high affinity for serum albumin, increasing its duration of action. Accordingly, its persistence as a potential antigen in circulation must not be ruled out. Glargine was created by modifying three amino acids. Two positively charged arginine residues were added to the C-terminus of the B-chain, and they shift the isoelectric point from 5.4 to 6.7, making Glargine more soluble at a slightly acidic pH and less soluble at a physiological pH. In order to avoid deamination it was necessary to replace the acid-sensitive asparagine at position 21 in the A-chain by glycine. These three structural changes and formulation with zinc result in a prolonged action when compared with biosynthetic human insulin. As it was speculated for Detemir, Glargine may induce antibodies, although in minimal proportion, due to aggregation at injection sites.

Glulisine is a rapid-acting insulin analog that differs from human insulin in that the amino acid asparagine at position B3 is replaced by lysine and the lysine in position B29 is replaced by glutamic acid.

Lispro was engineered through recombinant DNA technology so that the penultimate lysine and proline residues on the C-terminal end of the B-chain were reversed. This modification did not alter the insulin receptor binding, but blocked the formation of insulin dimers and hexamers. This allowed larger amounts of active monomeric insulin to be available for postprandial (after meal) injections. Similar to Glulisine, the monomeric state of Lispro theoretically may diminish the chance of phagocytosis by cells of the Immune System resulting in lower immunogenicity.

In order to express the degree of cross reactivity of IA induced by the initial insulin formulation (¨Ligand̈, L), against a panel of slow or fast action insulin analogs as described above (Competitors̈, C_i_), the parameter selectivity (S), as defined elsewhere (Berzofsky and Schechter [1]; Berzofsky et al. [7]), was employed. Approximate values for average (median) affinity (K_0_) for L and those from each C_i_, were obtained from the plots of B/F signals as a function of each competitor concentration, interpolating in the abscissa axis the respective doses corresponding to the maximum/2 signal [(B/F)_0_/2] (Graphical abstract, RIA plot). Alternatively, a best fit to the true affinity constants was obtained by representing B/F signals for each ligand as a function of free tracer (F) calculated by the formula: F = [C/(1 + B/F)]. It is important to emphasize that the molar concentration of the tracer in the test must be lower than the inverse of K_0_ value. This condition precludes the preparation of the respective labeled competitors to perform specific single RIAs for each homologous ligand. (Berzofsky and Schechter [1]). In the RIA the concentration of tracer was 5.8 × 10^−11^ M (see *Preparation of the homogeneous tracer mono [^125^I A14] Insulin*) and K_0_ values for IA (ranging 10^6^–10^8^ M^−1^) were obtained from previous work of our laboratory (Trabucchi et al. [8]; Trabucchi et al. [9]). Hence, for the RIA described here the condition of negligible concentration of tracer with respect to the inverse of affinity constant of insulin antibodies was satisfied with sufficient excess (3–5 orders of magnitude).

Finally, the respective Selectivity ratios were obtained using the expression: S_i_ = K_L_/K_Ci_.

## Results

From the first-line screening of circulating IA by RBA at the start of the study an atypical high value of B%: 30.5% was obtained, in accord with the difficulties reported during the insulin treatment of that patient. Due to the specific consultation made by the physicians involved about the possibility of changing treatment to an alternative hypoallergenic insulin, we suggested first testing in vitro a panel of available formulations against the same serum sample. For this purpose a RIA displacement experiment was carried out as detailed in Materials and Methods. The K_0_ values obtained from that study were similar for the regular human insulin originally administered to the patient and for Detemir (K_0_: 7.90 × 10^8^ M^−1^ and K_0_: 8.10 × 10^8^ M^−1^, respectively; hence Selectivity of IA for that insulin analog, S_Det_, was ∼1). On the other hand, the analogs Glargine and Lispro showed lower affinities (K_0_: 2.10 × 10^7^ M^−1^ and K_0_: 1.96 × 10^7^ M^−1^, respectively; S_Gla_: 7.90 × 10^8^ M^−1^/2.10 × 10^7^ M^−1^: 37.6; S_Lis_: 7.90 × 10^8^ M^−1^/1.96 × 10^7^ M^−1^: 40.3). Finally, the insulin analog Glulisine showed the lowest affinity (K_0_: 1.00 × 10^6^ M^−1^, S_Glu_: 7.90 × 10^8^ M^−1^/1.00 × 10^6^ M^−1^: 790). This means that Glulisine exhibited in vitro 1/790 IA binding rate compared with regular human insulin at the same doses. In consequence, we suggested that the analog Glulisine should be in principle the best theoretical option to replace conventional human insulin in order to minimize the new in vivo IA-insulin complexes formation in this patient. In agreement with such suggestion the replacement of NPH/regular insulin with this insulin analog Glulisine was made (0,6 IU/Kg/day by the pump infusion system, as indicated by Hoogma et al. [10]). After 6 months the patient recovered metabolic control, presenting with an HbA_1c_ of 7.6% (IFCC 60 mmol/mol), and a glucose level not exceeding 170 mg% (9.4 mmol/L). The IA evolution after 6 months of insulin change showed that the level varied from B%: 30.5 to B%: 27.0, and after 14 months of the new treatment the value dropped to B%: 17.4. A new RIA performed as a control on a serum sample obtained after 14 months showed a value of K_0_: 3.4 × 10^7^ M^−1^ for the analog Lispro, similar to the result obtained 14 months before, whereas the regular human insulin and the other insulin analogs tested as alternative competitive ligands exhibited very low immunoreactivity, preventing affinity calculations.

## Discussion

It was demonstrated that the analytical strategy presented here is a useful alternative to assess the in vitro antibody insulin interaction by applying a conventional RBA on serum samples from high IA responder patients as a first screening test and then measuring the degree of immune cross-reactivity exhibited by other possible replacement insulins. This approach is in accordance with the statements on the performance of the high sensitivity although “quasi-quantitative” immunochemical methods (RBA) compared with other “absolute” methods (RIA) which permit a more accurate and refined analysis of weaker cross-reactions (Mire-Sluis et al. [11]). Then, the application of a complete serial displacement RIA and data processing to calculate antibody K_0_ and S parameters on high titer IA sera potentially reactive against alternative insulin formulations, including insulin analogs, may be visualized as a rational and useful selection tool. In the case studied, the patient with initial high IA levels recovered a better metabolic control and exhibited acceptable laboratory values within a period of 6–14 months after the treatment with the selected insulin analog. The IA binding levels after 14 months of changing from regular and NPH human insulin to Glulisine, showed a significant decrease in B% signal and, in good accordance, the K_0_ parameters also decreased in all analogs except for Lispro. Although other explanations may not be ruled out, a feasible explanation for such metabolic improvement was that the selected analog Glulisine in fact presented less chance of capture by circulating IA and hence lower probability of in vivo new immune complex formation. Moreover, the persistent low IA levels during a period larger than one year, suggests that the selected insulin analog also caused *per se* minimal or negligible immune system specific restimulation.

Of course, large scale studies are necessary in order to demonstrate the actual benefits of this approach in support of eventual insulin schedule changes to ameliorate treatment of a high IA response in diabetic patients.

## Conclusion

From our preliminary results we conclude that conventional criteria for selection of insulin analogs (Thomas Danne-Jan Bolinder [12]), exclusively in terms of their pharmacokinetic and pharmacodinamic properties, should be reconsidered if a poorly controlled diabetic patient presents with high titers of IA. New evidence is necessary in order to demonstrate the actual benefits of this approach to ameliorate treatment of diabetic patients exhibiting undesirable high immunologic response to an initial hormonal replacement therapy.
